# Protective Effect of *T. violacea* Rhizome Extract Against Hypercholesterolemia-Induced Oxidative Stress in Wistar Rats

**DOI:** 10.3390/molecules17056033

**Published:** 2012-05-21

**Authors:** Olorunnisola Sinbad Olorunnisola, Graeme Bradley, Anthony Jide Afolayan

**Affiliations:** 1 Department of Biochemistry and Microbiology, University of Fort Hare, Private Bag X1314, Alice 5700, South Africa; E-Mails: sinbadkd@gmail.com (O.S.O.); GBradley@ufh.ac.za (G.B.); 2 Phytomedicine Research Group, Department of Botany, University of Fort Hare, Alice 5700, South Africa

**Keywords:** *T. violacea*, rhizomes, hypercholesterolemia, antioxidant enzymes lipid peroxidation

## Abstract

The present study examines the effect of methanolic extract of *T. violacea* rhizomes on high cholesterol (2%) diet fed rats (HCD). At the end of 4 weeks, serum total protein, albumin, reduced glutathione (GSH), and markers of oxidative stress viz., catalase (CAT), superoxide dismutase (SOD), thiobarbituric acid reactive substances (TBARS—a marker of lipid peroxidation), glutathione-S-transferase (GST) and glutathione peroxidase (GPx) in the serum, aorta, liver and heart of HCD and normal rats were assessed and compared. A significant (*p* < 0.05) elevation in TBARS, and a reduction (*p* < 0.05) in serum total protein, albumin, GSH and antioxidant enzyme activities was observed in tissues of HCD fed rats compared with the normal group. Co-administration of crude extracts of *T. violacea* rhizomes protected the liver, heart, serum and aorta against HCD-induced lipid peroxidation in a dose dependant manner. The activities of the extract (500 mg/kg) compared favorably with gemfibrozil. The extracts also protected against HCD-induced reduction in serum total protein, GSH and restored the activities of antioxidant tissues (liver, heart and aorta) enzymes to near normal values. This result suggested that consumption of *T. violacea* rhizome may help to protect against hypercholesterolemia- induced oxidative stress diseases in the heart and liver.

## 1. Introduction

Metabolic disturbances such as hyperlipidemia have been implicated in the etiopathogenesis of several human diseases. Dyslipidamia is viewed as the primary mediator of a cascade of heart damaging events such as acute pancreatitis (AP), renal injury [1,2], stroke, atherosclerosis [3] and metabolic syndrome [4]. It may be due to hereditary disorders [familial hypercholesterolemia (FH)] [5,6], polygenic hypercholesterolemia (familial combined hypercholesterolemia) or “non-lipid” diseases such as type 2 diabetes, cholestatic liver diseases, nephrotic syndrome, chronic renal failure, hypothyroidism, cigarette smoking and obesity [5,6]. Irrespective of the etiology of dyslipidemia, one common feature is elevated serum total cholesterol, low density lipoprotein, very low density lipoprotein (LDL) cholesterol, reduced high density lipoprotein (HDL) or elevated hypertriglyceridemia [7,8]. The current hypothesis suggests oxidative stress as an underlying mechanism by which dyslipidemia, particularly hypercholesterolemia, induces tissue damage or provokes several human diseases [9]. It was reported that hypertriglyceridemia and hypercholesterolemia may be responsible for oxidative modification of LDL, protein glycation, glucose-autooxidation with excess production of free radicals and lipid peroxidation products [10], which represent major risk factors for ischemic heart diseases [11]. A lot of studies have reported that increased aldehydes such as malondialdehyde (MDA) and conjugated dienes are involved in hyperlipidemia-provoked free radical attacks on membrane lipoproteins and polyunsaturated fatty acids [10]. Recently, there is growing body of scientific evidence of the protective biochemical functions of naturally occurring antioxidants in biological systems [12]. Antioxidants such as flavonoids, polyphenols, vitamin C and E and carotenoids have been reported to protect the body system against reactive oxygen species [13,14]. In the search for antioxidant compounds, various efforts are now concentrated on many herbal plant extracts because of their potential to induce antioxidant effects [15]. One of the plants commonly used in the Eastern Cape of South Africa in the treatment of diseases is *Tulbaghia violacea*. Rhizomes of *Tulbaghia violacea* were recently reported to be useful in the management of cardiovascular diseases [16]. Clear biochemical evidence indicates that *T. violacea* is endowed with significant *in vitro* antioxidant properties [17], anthelmintic activity [18] and anticancer properties [19], however, information about the *in vivo* antioxidant activity of the plant in diet-induced hypercholesterolemia is scanty in the literature. This study was designed to examine the effect of the methanolic extract of rhizomes of the plant on antioxidant enzyme status in hypercholesterolemic diet fed rats.

## 2. Results and Discussion

### 2.1. Total Protein and Albumin Contents

Evaluation of total protein or albumin status may be helpful in the assessment of disease progression [20]. Albumin is an important component of plasma antioxidant activity that primarily binds free fatty acids, divalent cations and hydrogen oxychloride (HOCI) [21]. The present study revealed significantly (*p* < 0.05) lower levels of total protein, albumin or albumin/globulin ratio in rats on high cholesterol diet when compared to animals with normal diet (Table 1). The decreased levels of total protein and albumin may be due to reduction in protein intake from the intestine as a result of a high calorie lipid diet [22], an indication of diminished synthetic function of the liver resulting probably from hepatocellular damage [23] or stress resulting from the increased metabolic need for tissue repair and free radical neutralization occasioned by the high fat diet [24]. However, co-treatment with extracts of *T. violacea* rhizomes or a standard drug (gemfibrozil) significantly restored the protein levels to near control levels. The activity of the extract is dose dependant and is comparable to the standard drug at the highest concentration (500 mg/kg). A similar observation had been previously reported after oral administration of avocado fruit pulp to hyperlipidemic rats [25]. The ability of the plant extract to protect against reduction in albumin or total protein resulting from a high cholesterol diet may be attributed to its free radical scavenging properties [17].

### 2.2. Lipid Peroxidation

The measurement of thiobarbituric acid (TBARS) is commonly used to monitor lipid peroxidation and indirectly, oxidative stress *in vitro* and *in vivo* [26]. Lipid peroxidation is initiated by free radical attack on membrane polyunsaturated fatty acids leading to their transformation and fragmentation to alkanes and reactive aldehyde compounds. Evaluation of the effect of high cholesterol diet in experimental rats showed a significant (*p* < 0.05) increase in TBARS levels in the liver, serum, heart, and aorta homogenates of HCD-fed rats compared to the normal group. The observed increase in lipid peroxidation (TBARS) in animals fed with high cholesterol is consistent with several clinical and experimental studies which have shown that hypercholesterolemia leads to increased lipid peroxidation [27]. Co-treatment of HCD fed rats with extracts of rhizome of *T. violacea* at a dose of 250 and 500 mg/kg significantly reduced the TBARS concentration (Table 2). The percentage reduction in all the tissues investigated [hepatic (16.34%, 35.20%), serum (22.0%, 28.4%) heart (31.0%, 36.8%) and aorta (18.9%, 24.4%)] was dose dependant and it increased with concentrations (Table 2). Also the percentage reduction caused by the extract (500 mg/kg. bwt) was comparable to that of gemfibrozil [hepatic tissue (36.56%), serum (29.9%), (heart 37.2%) and aorta (27.8%)]. The ability of the methanolic extract to inhibit the process of lipid peroxidation *in vivo* may be due to the free radical scavenging activities of its phytochemical components, as earlier reported by Olorunnisola *et al.* [17]. In addition, anti-lipid peroxidative activity of the extracts may be due to the presence of anti-lipidemic agents since activities were similar to those of the standard anti-lipidemic drug used in this research work (Table 2).

### 2.3. Glutathione Content in Liver, Serum, Heart and Aorta

Glutathione is a small tripeptide protein synthesized in the liver [28]. It is a potent antioxidant with high redox potential and it also serves as a co-factor for several oxidative stress detoxifying enzymes (glutathione peroxidase and glutathione transferase) [29,30]. Glutathione also helps in the regeneration of some important antioxidant vitamins such as C and E. Depletion of GSH has been reported in apoptosis and many degenerative conditions [30]. The results of the present study showed that the level of glutathione (GSH) was significantly decreased (*p* < 0.05) in the liver, serum, heart, and aorta homogenates of the animals fed with high-cholesterol diet compared to the control diet group. This observation is consistent with administration of high cholesterol in experimental rats [31]. It can be assumed that the reduction in tissue glutathione levels was as a result of increased oxidative stress and lipid peroxidation occasioned by the high cholesterol diet [32,33].

Co-administration to HCD-fed rats of extracts of rhizome of *T. violacea* at a dose of 250 and 500 mg/kg significantly prevented the reduction in the levels of GSH. The protective effect of the extracts is comparable with that seen with gemfibrozil (Table 2) and garlic, which belong to the same family [34,35]. It may be suggested that the activities of the plant are due to its free radical scavenging activities and the rich content in antioxidant phytochemical constituents (phenols and flavonoids) [17]. The ability of the extract to protect the heart and aorta against hypercholesterolemia-induced lipid peroxidation and oxidative stress may explain its folklore use in the management of cardiovascular diseases [17] and it may also serve a source of hepatoprotective agents.

### 2.4. Tissue Antioxidant Enzyme Defense System

High cholesterol diet has been reported to induce oxidative stress in various organs such as the liver, heart, and aorta [27]. It exerts is toxic effects by causing lipid peroxidation resulting in the formation of TBARS. Under normal conditions, antioxidant enzymes such as superoxide dismutase catalyse the conversion of superoxide radicals (O_2_^−^) into hydrogen peroxide (H_2_O_2_) and O_2_ [36] and catalase further detoxifies H_2_O_2_ into H_2_O and O_2_ [37] while glutathione peroxidase also functions in detoxifying H_2_O_2_ like catalase and GST plays an essential role in the liver by eliminating toxic compounds by conjugating them with GSH. However, imbalance between the formation of reactive oxygen species and their elimination occasioned by hypercholesterolemia has been implicated in oxidative-induced diseases.

**Figure 1:**
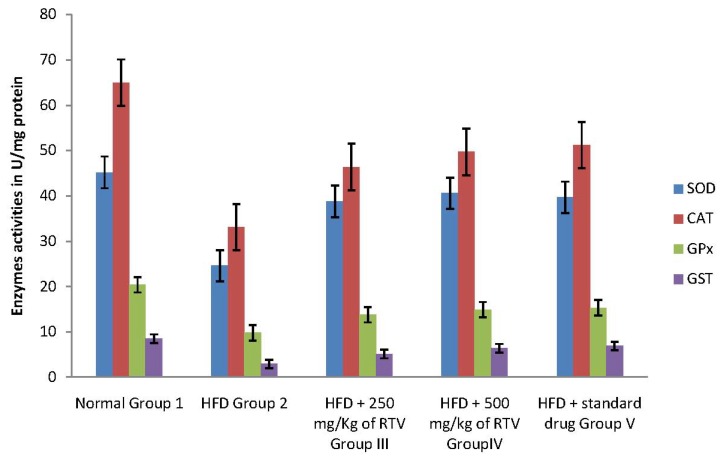
Effects of methanolic extract of rhizomes of *T. violacea* treatment on antioxidants defense viz., Superoxide dismutase (SOD), Catalase (CAT), glutathione peroxidase (GPx), and glutathione-S-transferase (GST) in high fat diet induced oxidative stress in rat liver homogenate.

**Figure 2:**
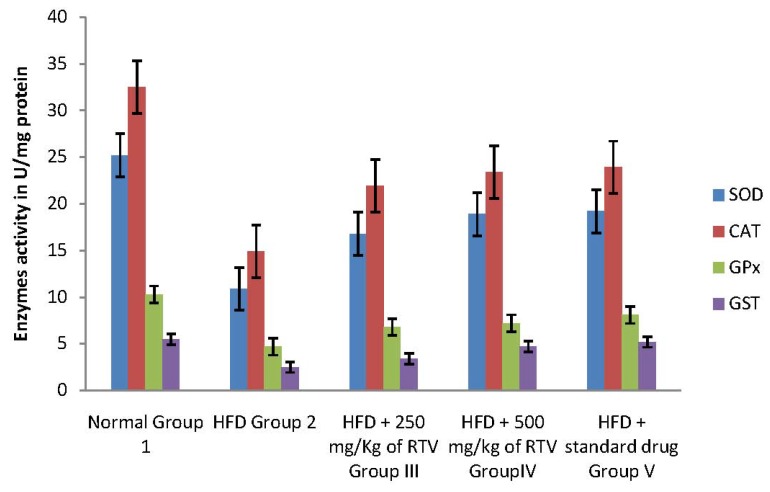
Effects of methanolic extract of rhizomes of *T. violacea* treatment on antioxidants defense viz., Superoxide dismutase (SOD), Catalase (CAT), glutathione peroxidase (GPx), and glutathione-S-transferase (GST) in high fat diet induced oxidative stress in rats aorta homogenate.

**Figure 3:**
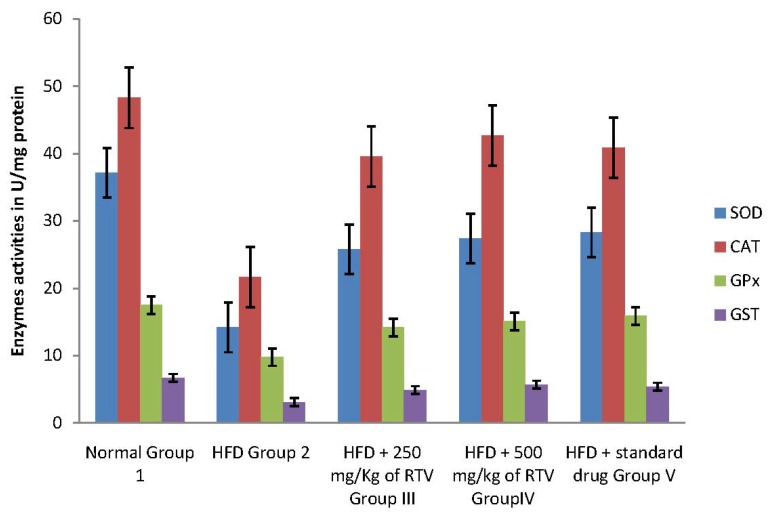
Effects of methanolic extract of rhizomes of *T. violacea* treatment on antioxidants defense viz., Superoxide dismutase (SOD), catalase (CAT), glutathione peroxidase (GPx) and glutathione-S-transferase (GST) in high fat diet induced oxidative stress in rats heart homogenate.

In the current study, hepatic, aorta and heart antioxidant enzymes (SOD, CAT, GPx and GST) activities significantly decreased in rats fed a cholesterol-rich diet compared to those fed a control diet (Figure 1, Figure 2 and Figure 3). The decrease in the activities of these enzymes could be attributed to the excessive utilization of these enzymes in inactivating the free radicals generated due to the high cholesterol diet [9] or insufficient availability of GSH. This observation is further substantiated by the elevated malondialdehyde levels (Table 2). Our results are in agreement with those of others, who studied the effect of high fat diet on liver [31], aorta [38] and heart [9] antioxidant enzyme systems, respectively. 

Co-administration to HCD-fed rats of extracts of rhizome of *T. violacea* at a dose of 250 and 500 mg/kg significantly prevented the reductions in the levels of SOD, GAT, GPx and GST in the liver, aorta and heart of rats in a dose dependant manner. The protective effect of the extracts is comparable with that of gemfibrozil (Table 2). Our results indicate that *T. violacea* had a free radical scavenging activity which probably provides organ protection from hypercholesterolemia. The reduction of serum and liver TBARS, and increased cardiac and aorta antioxidant enzyme activities in rats treated with *T. violacea* may be related to lipid-lowering ability because its activity compared favorably with gemfibrozil, a lipid lowering drug [39], or the presence of phenolic compounds.

It can also be hypothesized that the anti-oxidant enzyme in aorta, liver or heart may be up-regulated by administration of *T. violacea* in response to hypercholesterolemia-enhanced free radical production. A similar observation has been reported for cloves [*Syzygium aromaticum* (Gaertn) Linn.] a family of Myrtaceae [40], and garlic and onion extracts from the Alliacea family [40]. The extract also compared favourably with garlic oil, which was reported to increase hepatic glutathione S-transferase, glutathione reductase, superoxide dismutase [40]. However, contrary to garlic oil, extract of *T. violacea* increased the activity of glutathione peroxidase.

## 3. Experimental

### 3.1. Plant Collection and Extract Preparation

Plant collection and extract preparation was as described earlier by Mohammad and Woodward [41] and modified by Olorunnisola *et al*. [17]. 

### 3.2. Animals

Healthy, female, Wister albino rats (130–160 g) were randomly assigned to control and treated groups (six animals per group/cage). They were maintained under standard environmental conditions (22 ± 2 °C, 12:12 h dark/light cycle, humidity: 45–50%, frequent air change) and had free access to tap water and food. All animals were obtained from the animal house of the laboratory of the School of Biological Sciences, University of Fort Hare Alice 5700, South Africa. All procedures used in the present study followed the “Principles of Laboratory Animal Care” from NIH Publication No.85-23 and were approved by the Animal Ethics Committee of our university.

### 3.3. Experiment Design

After 2 weeks acclimation period, the experimental animals were divided into the following groups:

Group 1: Experimental animals fed with a standard diet and orally administered 1 mL distilled water served as control. 

Group II: Negative control fed with a high cholesterol diet and normal diet [standard diet + pure cholesterol (2% w/w)].

Groups III and IV: Fed with a high cholesterol diet orally [standard diet + pure cholesterol (2% w/w)] but also supplemented with extract of *T. violacea* orally (0.25 and 0.50 g/kg body weight, respectively) once daily for 4 weeks.

Group V: fed with high cholesterol diet [standard diet + cholesterol (2% w/w)] also supplemented with gemfibrozil orally (100 mg/kg body weight) once daily for 2 weeks. 

### 3.4. Preparation of Liver Homogenate

At the end of the treatment animals in all the groups were sacrificed using the method described by Yakubu *et al.* [42]. Briefly, under ether anesthesia the neck was quickly cleared of fur and skin to expose the jugular veins. These animals were thereafter bled through their cut jugular vein. The animals were quickly dissected and the liver removed. The liver was cleaned of blood and later blotted with a clean tissue paper, weighed and homogenized in 0.25 M sucrose solution (1:5 w/v) as described by Akanji and Yakubu [43]. The homogenate was later transferred to specimen bottles and kept frozen for 24 h at −4 °C before analyses. 

### 3.5. Heart Homogenate Preparation

Heart homogenate is prepared according to the method described by Noori *et al.* [44]. Briefly, the homogenate was prepared with ice in the ratio 4 g tissue for 16 mL of phosphate buffer (pH = 7.5) containing 1 mM/L Na_2_EDTA, 10 mL of 500 mM/L butylated hydroxytoluene (BHT) in acetonitrile was added to the prevent formation of new peroxides during the assay. The homogenates were centrifuged at 2,000 rpm for 4 min at 4 °C and the supernatant was frozen at −70 °C until analysis. 

### 3.6. Aorta Homogenate Preparation

Whole aorta tissue was homogenized in KH_2_PO_4_ buffer (100 mM) containing EDTA (1 mM) (pH 7.4, 1:10 w/v) and centrifuged (12,000 g, 30 min, 4 °C). The supernatant was used for biochemical estimations.

### 3.7. Biochemical Estimations

#### 3.7.1. Estimation of Total Protein and Albumin

Total protein was measured using the biuret reaction while albumin was measured by colorimetric estimation using the Sigma diagnostics albumin reagent (Sigma Diagnostics, Poole, UK) which contained bromocresol green (BCG). Globulin was obtained from the difference between total protein and albumin.

#### 3.7.2. Estimation of Malondialdehyde (MDA)

Thiobarbituric acids (TBARS) content, a measure of lipid peroxidation, was assayed in the form of Thiobarbituric Acid Reacting Substances (TBARS) according to Ohkawa *et al.* [45]. Briefly, a reaction mixture consisting of 8.1% sodium dodecyl sulphate (0.2 mL), 20% acetic acid solution (1.5 mL) adjusted to pH 3.5 with sodium hydroxide and 0.8% aqueous solution of thiobarbituric acid (1.5 mL) was added to 10% (w/v) of PMS (0.2 mL). The mixture was brought up to 4.0 mL with distilled water and heated at 95 °C for 60 min. After cooling with tap water, distilled water (1.0 mL) and the mixture of *n*-butanol and pyridine (15:1 v/v, 5.0 mL) was added and the mixture centrifuged. The organic layer was removed and its absorbance was measured at 532 nm and compared with those obtained from MDA standards. The concentration values were calculated from absorption measurements as standard absorption.

#### 3.7.3. Estimation of Glutathione Peroxidase

Glutathione peroxidase was measured by the method described by Rotruck *et al.* [46]. To Tris buffer (0.2 mL), ethylenediamine tetraacetic acid (EDTA, 0.2 mL), sodium azide (0.1 mL) and of tissue homogenate (Tris buffer 0.4 M, pH 7.0, 0.5 mL) were added. To the mixture, glutathione (GSH, 0.2 mL) followed by H_2_O_2_ (0.1 mL) were added. The contents were mixed well and incubated at 37 °C for 10 min, along with a control containing all reagents except tissue homogenate. After 10 min, the reaction was arrested by the addition of 10% trichloroacetic acid (TCA, 0.5 mL) and centrifuged. The activity was expressed as mg of GSH consumed/ min

#### 3.7.4. Determination of Superoxide Dismutase Activity

Superoxide dismutase was examined according the method of Misra and Fridovich [47]. Reaction mixtures contained sodium carbonate (1 mL, 50 mM), nitroblue tetrazolium (0.4 mL, 25 μm) and freshly prepared hydroxylamine hydrochloride (0.2 mL, 0.1 mM). The reaction mixtures were mixed by inversion followed by the addition of a clear supernatant of tissue homogenates (0.1 mL, 1:10 w/v). The change in absorbance of samples was recorded at 560 nm. 

#### 3.7.5. Estimation of Catalase

Catalase was assayed colorimetrically at 620 nm and expressed as moles of H_2_O_2_ consumed/min/mg protein as described by Sinha [48]. The reaction mixture (1.5 mL) contained 0.01 M pH 7.0 phosphate buffer (1.0 mL), tissue homogenate (0.1 mL) and 2 M H_2_O_2_ (0.4 mL). The reaction was stopped by the addition of dichromate-acetic acid reagent (2.0 mL, 5% potassium dichromate and glacial acid were mixed in 1:3 ratio).

#### 3.7.6. Estimation of GSH

GSH activity was determined by the procedure of [49]. The assay solution contained 10% BSA, 50 mM Phosphate buffer (pH = 7.6), 2 mM NADPH, 20 mM GSSG. Absorbance at 340 nm was recorded at a temperature of 25 °C. The activity was calculated the using the molar coefficient for NADPH of 6.22 μ·mol^−1^ × cm^−1^ and expressed in U/gm tissue.

#### 3.7.7. Determination of GST Activity

The activity of GST was determined according to the method of Habig *et al.* [50]. Briefly, CDNB solution (0.1 mL) was pipetted into a conical flask before adding phosphate buffer (1 mL) and distilled water (1.7 mL). Next, the mixture was incubated at 37 °C for 5 min. The serum sample (0.1 mL) and GSH solution (0.1 mL) were added (using an automatic micropipette) after the incubation. A blank devoid of the serum was prepared for background correction. Absorbance readings at 340 nm were taken for 5 min at 60 s interval using a UV-VIS Analyst spectrophotometer. 

### 3.8. Statistical Evaluation

Statistical evaluation was done using one-way analysis of variance (ANOVA) followed by Duncan’s Multiple Range Test. Statistical significance was set at (*p* < 0.05).

## 4. Conclusions

The present study clearly shows that rhizomes of *T. violacea* may be of therapeutic importance, not only as an antioxidant agent but also as a cyto-protective agent to protect the liver, aorta and cardiac injury from hypercholesterolemia.

## Figures and Tables

**Table 1:** Effects of crude extract of rhizomes of *T. violacea* on total protein, albumin, and globulin protein of rats kept on a high-fat diet.

Treatment group	Total protein (mg/dL)	Albumin (mg/dL)	Globulin (mg/dL)	Albumin / globulin ratio
Normal	7.41 ± 0.12	3.82 ± 0.38	3.01 ± 0.11	1.26
HCD only	6.62 ± 0.14 ^a^	3.31 ± 0.41 ^a^	3.31 ± 0.14	1.00 ^a^
HCD + 250 mg/kg RTV	6.92 ± 0.14	3.60 ± 0.43	3.32 ± 0.13	1.08
HCD + 500 mg/kg RTV	7.06 ± 0.19	3.79 ± 0.39	3.27 ± 0.12	1.16
HCD + 50 mg/kg Gemfibrozil	7.29 ± 0.13	3.84 ± 0.33	3.45 ± 0.19	1.11

Values are mean ± standard error of mean of six Wistar rats. HCD-Represents high cholesterol diet, RTV represents rhizomes of *Tulbaghia violacea* and ^a^ = (*p* < 0.05).

**Table 2:** Effects of crude extract of rhizomes of *T. violacea* on thiobarbituric acids (TBARS) and reduced glutathione (GSH) in tissues of rats kept on a high-cholesterol diet.

Group	NC	HCD	HCD + 250 mg/kg of RTV	HCD + 500 mg/kg of RTV	HCD + Gemfibrozil
Liver					
TBARS	2.75 ± 0.12	4.65 ± 0.19 ^a^	3.89 ± 0.14 ^b^	3.01 ± 0.16 ^b^	2.95 ± 0.11 ^b^
GSH	5.32 ± 0.17	3.12 ± 0.13 ^a^	4.22 ± 0.10 ^b^	4.89 ± 0.14 ^b^	5.01 ± 0.18 ^b^
Serum					
TBARS	11.2 ± 0.13	17.45 ± 1.14 ^a^	13.65 ± 0.11 ^b^	12.50 ± 0.15 ^b^	12.41 ± 0.18 ^b^
GSH	1.23 ± 0.11	0.19 ± 0.12 ^a^	0.89 ± 0.17 ^b^	1.02 ± 0.13 ^b^	1.01 ± 0.31 ^b^
Heart					
TBARS	8.52 ± 1.12	14.25 ± 0.17 ^a^	9.85 ± 0.21 ^b^	9.01 ± 1.10 ^b^	8.95 ± 0.12 ^b^
GSH	4.85 ± 0.20	2.15 ± 0.28 ^a^	3.19 ± 0.15 ^b^	3.39 ± 0.24 ^b^	3.97 ± 0.13 ^b^
Aorta					
TBARS	10.5 ± 1.32	15.23 ±0.52 ^a^	12.35 ± 0.15 ^b^	11.51 ± 0.16 ^b^	10.99 ± 0.20 ^b^
GSH	8.25 ± 1.70	4.82 ± 0.19 ^a^	6.98 ± 0.17 ^b^	7.21 ± 0.13 ^b^	7.37 ± 0.10 ^b^

Values are mean ± standard error of mean of six Wistar rats. Significant changes are shown by ^a^ and ^b^ = (*p* < 0.05).
